# Development and Validation of a Clinlabomics‐Based Nomogram for Predicting the Prognosis of Small Cell Lung Cancer in China: A Multicenter, Retrospective Cohort Study

**DOI:** 10.1002/cam4.71180

**Published:** 2025-08-27

**Authors:** Qi Peng, Fang Yang, Ke Xu, Wei Guo, Dongsheng Wang, Mingfei Xiang, Huaichao Luo

**Affiliations:** ^1^ Department of Clinical Laboratory, Sichuan Clinical Research Center for Cancer, Sichuan Cancer Hospital & Institute, Sichuan Cancer Center University of Electronic Science and Technology of China Chengdu China; ^2^ Department of Oncology The First Affiliated Hospital of Chengdu Medical College Chengdu China; ^3^ Medical Insurance Division, Sichuan Clinical Research Center for Cancer, Sichuan Cancer Hospital & Institute, Sichuan Cancer Center University of Electronic Science and Technology of China Chengdu China

**Keywords:** clinlabomics, multicenter, prognosis, SCLC

## Abstract

**Background and Objective:**

Small cell lung cancer has a high incidence and mortality rate, frequently metastasizes, and is associated with a poor prognosis. However, traditional prognostic models based on stage alone cannot meet clinical needs. This study aims to establish a clinlabomics‐based, highly accessible prognostic model for small cell lung cancer.

**Methods:**

We conducted a multicenter observational retrospective study, enrolling clinical laboratory data of 276 small cell lung cancer patients. The cohort from Sichuan Cancer Hospital comprised a total of 196 samples. Of these, 88 samples were designated as the internal validation set, while 80 samples from an alternate institution were allocated as the external independent validation set. Utilizing the log‐rank test, univariate and multivariate Cox regression analyses, six prognostic indicators were discerned. A nomogram was subsequently developed based on these identified indicators.

**Results:**

Through the log‐rank test, univariate and multivariate Cox regression analyses, total protein (TP) (HR = 0.47, *p* < 0.001), aspartate aminotransferase (AST) (HR = 1.82, *p* < 0.001), and lymphocyte ratio (Lym ratio) (HR = 0.47, *p =* 0.005) were identified as laboratory biomarkers related to prognosis from 61 blood‐related laboratory tests, covering routine blood, biochemical, coagulation, and infectious disease markers, while age, stage, and smoking were identified as clinical independent prognostic factors. A nomogram was developed based on these six indicators. The AUC of time‐independent ROC for 2‐ and 3‐year overall survival (OS) was 0.74, 0.74 in the training cohort, and 0.64, 0.74 in the validation cohort, respectively. The novel nomogram accurately predicted the prognosis for two independent cohorts with *p* values < 0.001 and performed risk adjustment, which classified patients with different OS at the same extensive stage (ES) or limited‐stage (LS).

**Conclusions:**

The clinlabomics‐based nomogram helps to more effectively predict the prognosis of small cell lung cancer by leveraging blood laboratory data.

## 1 Introduction

Small cell lung cancer (SCLC) is an aggressive neuroendocrine cancer with 25,000 new cases per year globally, a high mortality rate causing at least 200,000 deaths per year, and has an extremely poor survival rate [1, 2]. SCLC is a highly aggressive form of cancer, with nearly 67% of patients presenting with distant metastasis at the time of diagnosis, and a 5‐year survival rate of less than 7% [3].

SCLC is clinically divided into limited‐stage (LS) and extensive‐stage (ES) based on metastatic extent. LS corresponds to TNM stages I–IIIB, while ES involves contralateral lung and distant metastases (stage IV) [4]. Treatment for LS typically includes cisplatin and etoposide with radiotherapy [5], achieving a 3‐year survival rate of 14.3% [6]. Prophylactic cranial irradiation (PCI) can further increase 3‐year overall survival (OS) by 5.4% in responders [7]. ES patients receive chemotherapy combined with immunotherapy, such as platinum‐etoposide plus durvalumab [8], with a 1‐year survival rate of 30%–40% [9]. However, prognosis varies widely among patients, and relying solely on stage for prognosis is inaccurate. Additional independent prognostic factors are needed. Notably, over 90% of SCLC patients have a smoking history [10], which is associated with increased treatment toxicity and shorter survival in ES disease [11]. Furthermore, genetic mutations are also an important factor affecting patient prognosis. Amplification of the genomic region containing the KDR, PDGFRA, and KIT genes on 4q12 is associated with increased survival in patients, whereas inactivation of the Wnt pathway regulator APC and amplification of the CCNE1 gene are associated with worse prognosis [12]. In recent years, the application of liquid biopsy has provided new directions for the development of tumor biomarkers. In SCLC, the methylation of SHOX2 and RASSF1A has been confirmed to be associated with prognosis and can serve as potential diagnostic and prognostic biomarkers [13]. Additionally, miR‐200b‐3p, miR‐3124‐5p, and miR‐92b‐5p have also been identified as potential diagnostic and prognostic biomarkers for SCLC [14]. Furthermore, exosomes have been reported to interact with immune cells such as CD8‐positive T cells and natural killer (NK) cells, promoting tumor growth and demonstrating clinical potential as prognostic biomarkers [15]. However, the detection of genetic mutations and the application of liquid biopsy are affected by high research and development costs, which result in high prices and long turnaround times. Therefore, it will still take some time for these technologies to be widely promoted.

Clinical laboratories generate a large amount of data every day. Multiple studies have demonstrated that clinical laboratory indicators are associated with tumor prognosis. Elevated serum AST levels are associated with poor prognosis in non‐small cell lung cancer (NSCLC) patients and recurrence after liver cancer surgery [16, 17]. Changes in lymphocyte ratio can serve as a potential prognostic biomarker for tumors [18]. The previous study has shown that RDW is an independent risk factor for cancer‐specific survival in CRC patients [19]. Platelets in cancer patients have also been proven to play a crucial role in the microenvironment, thereby affecting patient prognosis [20, 21]. Another study for small cell lung cancer indicated that high levels of LDH and NSE, as well as NLR (neutrophil–lymphocyte ratio), were associated with poor prognosis in patients [22]. In early esophageal cancer, high levels of NLR and MLR were associated with submucosal tumor invasion in EEC patients [23]. Our group also discovered that peripheral blood IMR prognostic index can be used as an independent predictor of SCLC patients before treatment [24]. In summary, the aforementioned literature indicated that certain laboratory indicators could serve as prognostic factors. However, there has been no systematic assessment of the prognostic value of all the laboratory indicators. Previously, our team introduced ‘clinlabomics’, a concept leveraging routine laboratory data to address numerous clinical issues effectively [25]. Based on the methods of this concept, a study have discovered new phenotypes of acute ischemic stroke, diagnosing retinal detachment and brain metastasis of lung cancer [26, 27, 28], demonstrating the feasibility of using clinlabomics to solve clinical problems. Therefore, this study employed clinlabomics in an attempt to address the lack of efficient and accessible prognostic models for small cell lung cancer.

Through comprehensive evaluation of clinical biochemical markers, routine blood tests, coagulation profiles, and infectious disease data in small cell lung cancer (SCLC) patients, we identified AST (aspartate aminotransferase), TP (total protein), and lymphocyte ratio as independent prognostic factors using log‐rank testing, univariate, and multivariate analyses. Combining this information with clinical and pathological data, we have successfully developed and validated a novel nomogram. This nomogram allows for a more precise prediction of the prognosis for SCLC patients. Once a patient was diagnosed with SCLC, clinicians can rapidly identify whether the patient belongs to the high‐risk group with poor prognosis by inputting blood indicators and clinical pathological information obtained at admission into the nomogram model, thereby providing a reference for clinical treatment planning.

## 2 Results

### 2.1 Baseline Characteristics of 276 Patients With SCLC

In this study, the clinical information included age, gender, smoking, stage, follow‐up duration, and survival status were collected. The distribution of data from Sichuan Cancer Hospital (SCCH) and The First Affiliated Hospital of Chengdu Medical College is presented in Tables 1 and 2. The median age of patients at both centers exceeded 60 years, with the median age being 60.5 years (range: 28–79 years) in SCCH and 66 years (38–81 years) in another hospital. Additionally, the proportion of smokers was higher than that of non‐smokers at both institutions, with 58.2% and 68.8% respectively. Male patients outnumbered female patients, with males comprising 71.9% and females 28.1% in SCCH, and 71.2% male and 28.8% female in the other cohort. During the follow‐up period, 66.8% of the patients died, with a median survival time of 17.55 months (range: 0.3–80.8 months). At the other center, 81.2% of the patients were deceased by the end of the follow‐up period, with a median survival time of 7.5 months (range: 1–66 months). The staging at presentation varied between the centers. In SCCH, a greater proportion of patients were in the LS than the ES, with 72.4% in LS and 27.6% in ES. In contrast, at the other cohort, fewer patients were in LS compared to ES, with the proportions being 40% and 60%, respectively. In an external independent cohort of 80 patients, the median values for TP, AST, and lymphocyte ratio were 66.75 (range: 50.40–78.60), 24 (range: 10.0–193.0), and 18.10 (range: 3.90–44.90), respectively. The details on the laboratory indices data of 196 patients in SCCH could be found in the Table S2.

**TABLE 1:** The clinical characteristics of 196 patients with SCLC in SCCH.

Clinical characteristics	Number (%)/median (range)	
Age	60.5	(28–79)
Gender		
Male	141	(71.9)
Female	55	(28.1)
Smoking history		
Nonsmoking	82	(41.8)
Smoking	114	(58.2)
Event		
Alive	65	(33.2)
Dead	131	(66.8)
Stage		
I–III	142	(72.4)
IV	54	(27.6)
Time (month)	17.55	(0.3–80.8)

**TABLE 2:** The clinical characteristics of 80 patients with SCLC in external independent validation cohort.

Clinical characteristics	Number (%)/median (range)	
Age	66.5	(38–81)
Gender		
Male	57	(71.2)
Female	23	(28.8)
Smoking history		
Nonsmoking	25	(31.2)
Smoking	55	(68.8)
Event		
Alive	15	(18.8)
Dead	65	(81.2)
Stage		
I–III	32	(40.0)
IV	48	(60.0)
Time (month)	7.5	(1–66)
TP (g/L)	66.75	(50.40–78.60)
Lymphocyte ratio (%)	18.10	(3.90–44.90)
AST(U/L)	24	(10.0–193.0)

### 2.2 Selection of Six Predictive Indicators

**FIGURE 1:**
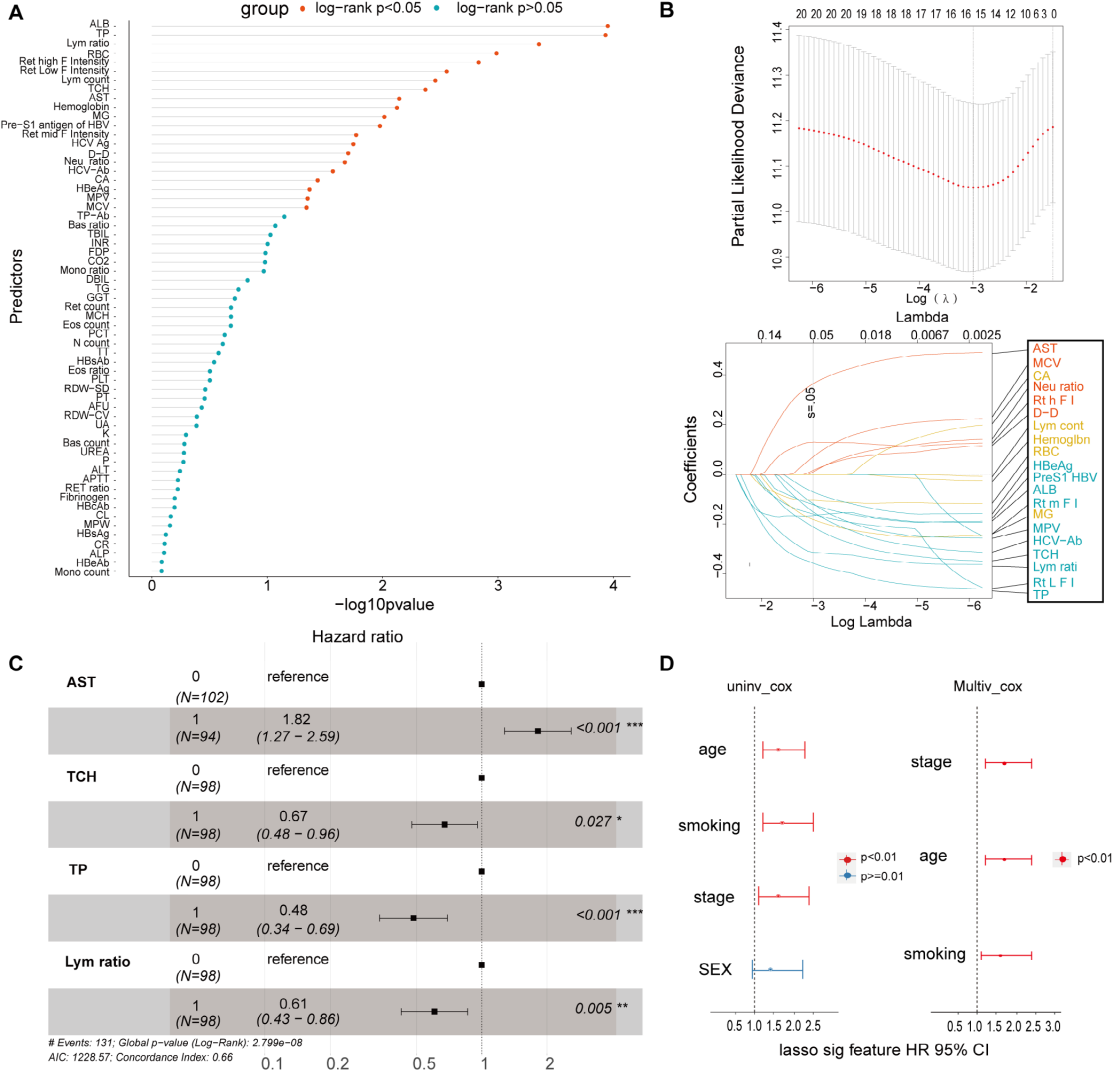
Selection of six predictors. (A) 61 predictors in clinical laboratory data of SCC patients. (B) Lasso regression for 21 risk factors. (C) Ggforest for four prognostic candidates. (D) Univariate and multivariate Cox regression for clinical pathological data.

We conducted prognostic analysis on 61 laboratory indicators, including 23 routine blood indicators, 22 clinical biochemical indicators, 7 coagulation function indicators, and 9 infectious disease indicators for 196 small cell lung cancer patients in SCCH, and 21 markers were found to be significant (Figure 1A). Subsequently, lasso‐cox regression was used to select the best predictive factors, and among them, 16 candidates had the minimum lambda value (Figure 1B and Table 3). As shown in Table 3, separate Cox regression analyses were performed for each of the 16 prognostic indicators for survival. We obtained eight indicators with *p* values < 0.01, among which six indicators with HR < 1 were considered protective factors for SCLC, namely ALB (HR = 0.51, 95% CI: 0.36–0.72, *p* < 0.001), TP (HR = 0.51, 95% CI: 0.36–0.72, *p* < 0.001), lymphocyte ratio (HR = 0.54, 95% CI: 0.398–0.77, *p* < 0.001), RBC (HR = 0.56, 95% CI: 0.4–0.8, *p =* 0.0012), Ret low F intensity (HR = 0.59, 95% CI: 042–0.84, *p* = 0.0031), TCH (HR = 0.61, 95% CI: 0.43–0.86, *p =* 0.0047), while the other two indicators with HR > 1 were AST (HR = 1.6, 95% CI: 1.1–2.3, *p =* 0.0077), Ret high F intensity (HR = 2.0, 95% CI: 1.3–3.1, *p =* 0.0018), respectively. Subsequent multivariate analysis revealed that TP (HR = 0.6, 95% CI: 0.4–0.89, *p* = 0.012), lymphocyte ratio (HR = 0.67, 95% CI: 0.46–0.96, *p* = 0.028), TCH (HR = 0.68, 95% CI: 0.48–0.96, *p* = 0.03), and AST (HR = 1.7, 95% CI: 1.2–2.4, *p* = 0.0049) were independent risk factors for OS. Further Cox regression was applied to filter these four markers. The indices AST (*p* < 0.001), lymphocyte ratio (*p* < 0.001), TP (*p =* 0.005) had *p* values less than 0.01, and therefore were selected for subsequent modeling (Figure 1C). We also performed prognostic analyses on four clinical variables: gender, age, smoking, and stage. Ultimately, age (HR = 1.6, 95% CI: 1.2–2.3, *p* = 0.006), smoking (HR = 1.7, 95% CI: 1.2–2.5, *p =* 0.0031), and stage (HR = 1.6, 95% CI: 1.1–2.4, *p =* 0.009) were identified as independent prognostic factors (Figure 1D).

**TABLE 3:** Univariate and multivariate Cox proportional hazards regression analysis of OS.

Variable	Univariate		Multivariate	
	HR (95% CI)	*p*	HR (95% CI)	*p*
ALB	0.51 (0.36–0.72)	**< 0.001**	0.78 (0.52–1.2)	0.24
TP	0.51 (0.36–0.72)	**< 0.001**	0.6 (0.4–0.89)	**0.012**
Lymphocyte ratio	0.54 (0.38–0.77)	**< 0.001**	0.67 (0.46–0.96)	**0.028**
RBC	0.56 (0.4–0.8)	**0.0012**	0.81 (0.55–1.2)	0.28
Ret Low F Intensity	0.59 (0.42–0.84)	**0.0031**	0.74 (0.5–1.1)	0.13
TCH	0.61 (0.43–0.86)	**0.0047**	0.68 (0.48–0.96)	**0.03**
MG	0.63 (0.45–0.9)	0.01		
PreS1 antigen of HBV	0.64 (0.45–0.9)	0.011		
HCV Ab	0.66 (0.45–0.6)	0.028		
HBeAg	0.68 (0.46–0.99)	0.044		
MPV	0.7 (0.5–0.99)	0.046		
MCV	1.4 (1.0–2.0)	0.047		
AST	1.6 (1.1–2.3)	**0.0077**	1.7 (1.2–2.4)	**0.0049**
D‐D	1.5 (1.1–2.1)	0.021		
Neu ratio	1.5 (1.1–2.1)	0.022		
Ret high F Intensity	2 (1.3–3.1)	**0.0018**	1.3 (0.76–2.1)	0.37

*Note:* *p*‐values less than 0.05 after multivariate analysis are bolded. Corresponding univariate *p*‐values for these indicators are also bolded.

### 2.3 Construction of the Nomogram With Six Indicators

**FIGURE 2:**
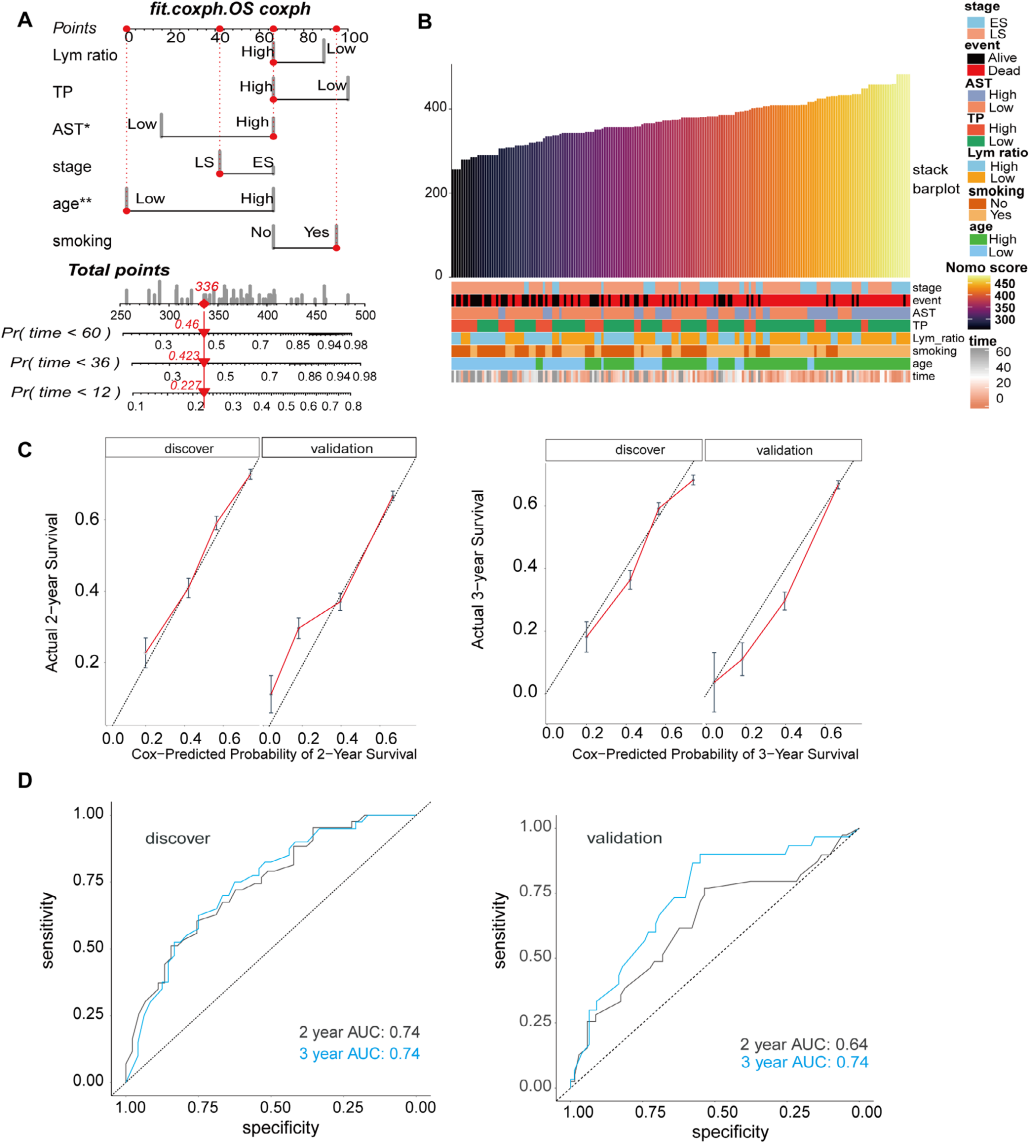
Construction and validation of a nomogram. (A) Nomo scoring criteria and the correspondence between total score and possibility of < 1, 3, 5 years for OS. (B) Heatmap of 196 patients with different nomo scores and clinical features. The color closer to bright yellow indicates a higher nomogram score, while the color closer to dark blue or black indicates a lower nomogram score. (C) Calibration curves for the discovery and validation groups for predicting 2‐year (left) and 3‐year (right) OS. (D) Receiver operating characteristic (ROC) curves for 2‐ and 3‐year survival based on the nomogram constructed in discover cohort (left) and validation cohort (right). AUC, area under the curve.

Scores were assigned to these six independent prognostic factors based on the Cox regression, and this prognostic model was created in discovery cohorts. Elevated levels of AST, staging at the ES, older age, and a history of smoking were associated with a higher probability of poor prognosis. SCLC patients with AST levels higher than 25 U/L scored 66, while those with lower levels scored 16. Smoking scored 95, compared to 66 for non‐smokers. Those in extensive stage scored 66, while those in the limited stage scored 42. Patients older than 58.5 years scored 66, whereas those younger scored 0. Conversely, high levels of blood TP and the lymphocyte ratio are beneficial for patient survival. Patients with TP content above 70.4 g/L scored 66, while those with lower levels scored 100. The lymphocyte ratio higher than 22.9% scored 66, and those with lower levels scored 89 (Figure 2A). According to these score criteria, we converted the laboratory data clinical pathological information of all 196 patients into nomogram scores and reorganized them based on the score levels (Figure 2B). As shown in the figure, the majority of patients with high nomogram scores were older, and had elevated AST levels, a more advanced stage, lower levels of lymphocyte ratio and T and P, a history of smoking. In addition, patients with dead events were mainly concentrated in the high‐score segments, and they also had shorter survival time, which indicated that SCLC patients with different ages, stages, and laboratory data could be distinguished partly by nomogram score. The independent 80 cohort performed the nomogram score transformation as well. Patients with different clinical characteristics and outcomes were also discriminated (Figure S1B).

### 2.4 Validation of Nomogram Model

For 2‐year survival rates, the predicted values in the discovery group were close to the actual values. However, in the validation group, while the prediction for patients with higher survival rates was consistent, the predicted OS for patients with poor prognosis was lower than the actual value. For 3‐year survival rates, the training group's predictions for patients with worse prognosis were more accurate than those for patients with better prognosis, while in the validation group, the predictions were more accurate for both patients with good and poor prognosis, but the fit with the predicted values was not high for the middle portion (Figure 2C). Overall, it was observed that the cox predicted probability was generally consistent with the actual value. Besides, time‐dependent receiver operating characteristic (time‐ROC) was utilized to evaluate the predictive accuracy of the nomogram. In the discovery and validation cohorts, the AUC for 2‐ and 3‐year OS were 0.74, 0.74 and 0.64, 0.74, respectively (Figure 2D). As regards the external independent center, the AUC for 2 and 3 years were even better, with 0.92 and 0.82 respectively (Figure S1A).

### 2.5 Comparison of Predictive Accuracy Between Novel Model and Previous Stage‐Based Prognostic System

**FIGURE 3:**
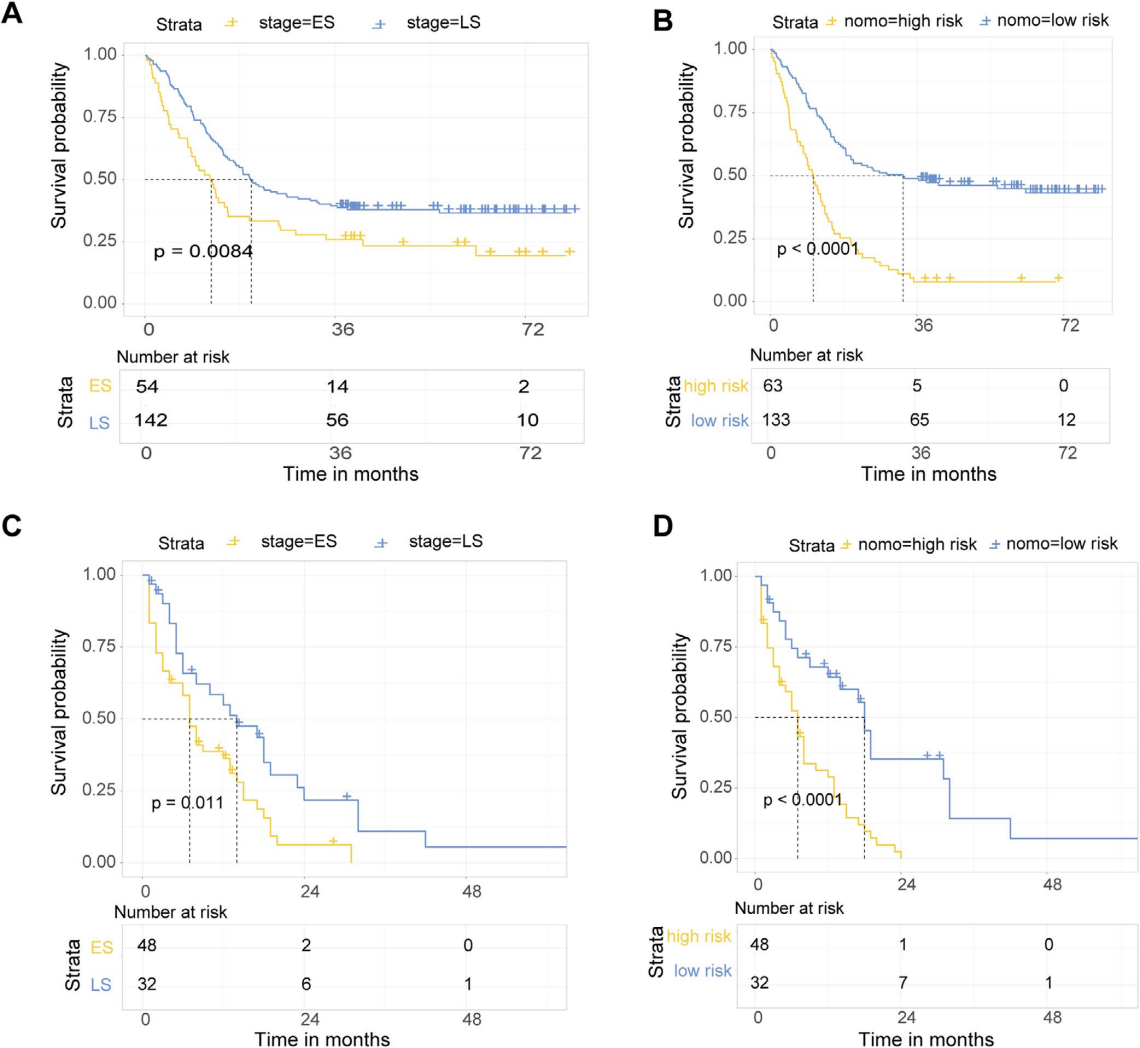
Comparison of predictive accuracy between novel and previous models. (A, C) Traditional prognostic models of multi‐center cohorts. (B, D) Novel nomograms predict the prognosis for groups with high risk and low risk.

The cutoff value of the discover group was determined based on the scoring system, with a value of 404.5. This system was used to stratify 196 SCC patients and independent cohort of 80 into high‐risk and low‐risk groups. The novel predictive model allowed for more accurate differentiation among patients with varying survival periods. The prognosis model based on stage identified 54 individuals in ES and 142 in LS. The number of survivors at 3 years was 14 for ES and 56 for LS. The 6‐year survival rates were 3.7% for ES and 7% for LS (HR = 1.6, 95% CI: 1.1–2.4, log‐rank *p* = 0.0084). In contrast, stratification through the nomogram score delineated groups into high‐risk (63 individuals) and low‐risk (133 individuals). At 3 years, the survival figures stood at 5 and 65 individuals for high‐risk and low‐risk groups, respectively, whereas the five survival numbers were 0 and 12 individuals, respectively (HR = 2.96, 95% CI: 2.08–4.21, log‐rank *p* < 0.0001). The new model has thus made corrections to survival rates across different periods, exhibiting statistically more significant implications (Figure 3A,B). In the independent cohort, a similar trend was observed where the hazard ratio for the stage prognostic model was 2.0 (95% CI: 1.16–3.45), with a log‐rank *p* value of 0.011, whereas the HR for the nomogram score group was 3.22 (95% CI: 1.82–5.88), with a log‐rank *p* value less than 0.0001(Figure 3C,D), which indicated that the nomogram improved survival predictions for SCLC patients.

### 2.6 Novel Model Distinguished Risk Stratification for SCLC Patients With the Same Stage

**FIGURE 4:**
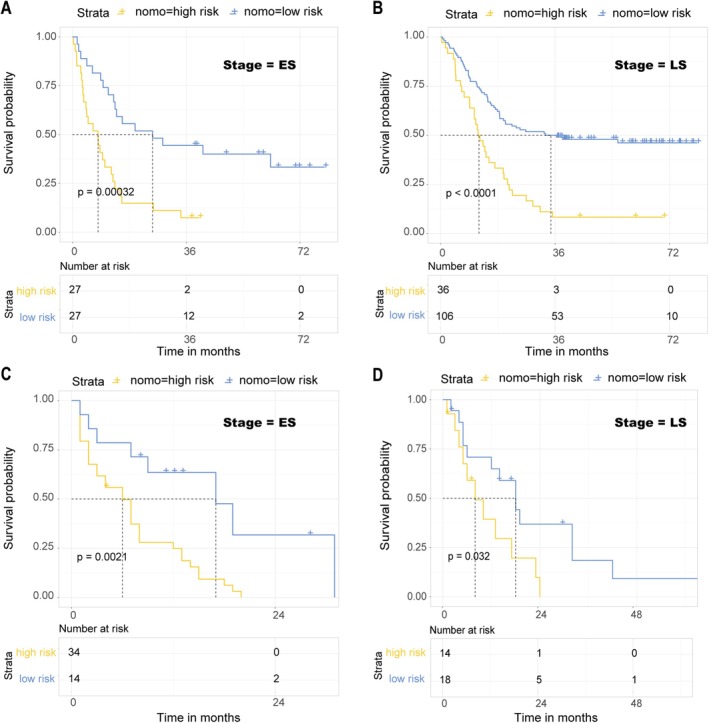
Novel models separated prognosis of patients within same stages. Novel prognostic model accurately predicts the outcomes of ES (A) and LS (B) patients in the SCCH cohort and the independent cohort (C, D).

According to the nomo score system, we categorized SCLC patients in ES into high‐risk and low‐risk groups and conducted an analysis of overall survival periods. The OS for 3 years in the high‐risk group was 7.4%, and the 6‐year OS was 0, which was significantly lower than the 44.4% and 7.4% for the low‐risk group (HR = 3.12, 95% CI: 1.64–5.88, log‐rank *p =* 0.00032) (Figure 4A). As for patients in LS, the 3‐ and 6‐year survival rates for the high‐risk group were 8.3% and 0%, respectively, compared to 50% and 9.4% for the low‐risk group (HR = 2.70, 95% CI: 1.75–4.17, log‐rank *p* < 0.0001) (Figure 4B). In the independent cohort, ES patients had poorer survival rates, with the longest survival time being 31 months. After grouping according to the nomo score, we were able to distinguish patients in ES with different outcomes (HR = 3.33, 95% CI: 1.47–7.69, log‐rank *p* = 0.0021). Thirty‐four individuals were categorized into the low‐risk group, of whom two survived over 24 months, while none of the 14 high‐risk patients survived beyond 24 months (Figure 4C). Patients in LS exhibited the same trend, with the overall survival rate of the low‐risk group being higher than that of the high‐risk group (HR = 2.44, 95% CI: 1.03–5.88, log‐rank *p* = 0.032). In the low‐risk group, five people survived over 2 years and one for more than 4 years, while in the high‐risk group, only one person survived for 24 months (Figure 4D).

## 3 Discussion

To develop a prognostic model for SCLC based on clinical laboratory indicators, we systematically analyzed 61 indicators from 196 patients with SCLC, identifying three independent diagnostic indexes: lymphocyte ratio, TP, and AST. These indicators were combined with three clinical indicators—age, smoking, and stage—to construct the prognostic model. The model's accuracy was validated by an external independent validation group consisting of 80 individuals. Our new model not only provides more accurate predictions than previous models but also differentiates between patients at the same stage with different outcomes.

Earlier researches have demonstrated that systematic assessment of blood laboratory data could provide significant insights into disease diagnosis, typing, and prognostic evaluation. Integrating peripheral blood laboratory data using machine learning can significantly improve the diagnosis of sepsis [29] and the prognostic assessment of myocardial infarction [30]. In addition, applying systematic analysis of laboratory data combined with clinical information can also enable more accurate diagnosis [31], prognosis, and subtype differentiation for ovarian cancer [32]. Another study demonstrated that analyzing clinical and laboratory data through a machine learning model can provide a more accurate diagnosis for the early stage of NSCLC than either standard eligibility criteria for screening or the mPLCOm2012 [33]. To sum up, whether it is viral infections, tumors, or non‐tumor diseases such as myocardial infarction and sepsis, systematic laboratory data analysis and integration can provide earlier and more accurate judgments for disease diagnosis and prognosis, aiding clinical decision‐making.

Briefly revisiting the three diagnostic indicators we identified—TP, AST, and lymphocyte ratio—it has been reported in previous studies that they are associated with tumor prognosis. Prior reports have indicated that preoperative AST levels were associated with prognosis in non‐small cell lung cancer patients [34] and esophageal squamous cell carcinoma [35]. In a phase III clinical trial, an increased level of AST was an adverse event that led to the discontinuation of pazopanib treatment in patients with locally advanced renal cell carcinoma (RCC) [36]. As for lymphocyte ratio, it has been demonstrated as an independent prognostic indicator for locally advanced nasopharyngeal carcinoma [37] and improved prognosis was related to increased tumor‐infiltrating lymphocytes in patients who have SCLC with neurologic paraneoplastic syndromes [38]. The underlying mechanism may be related to the tumor microenvironment. Tauriello DVF and colleagues revealed decrease of lymphocyte infiltration in the tumor microenvironment leading to colon cancer metastasis was an important reason for poor outcome [39]. Moreover, lymphocytopenia caused by radiotherapy also affects overall survival of lung cancer patients [40]. These studies suggest that lymphocyte ratio is an important factor affecting the prognosis of tumor patients. Not only the lymphocyte ratio but also the serum TP was important for the prognosis of tumor patients. Cancer patients usually had anorexia, accompanied by decreased protein intake, becoming significant predictors of mortality [41]. Postoperative liver dysfunction was associated with poor long‐term prognosis in patients with stage III colon cancer [42]. Considering that TP levels can fluctuate due to recent diet, inflammation, age, and other factors, both cohorts in this study are from the same region with minimal dietary differences. Therefore, our prognostic model may have limitations. If the prognosis involves cross‐regional analysis, it may be necessary to include more patients from different regions in future studies for calibration. In conclusion, the aforementioned literature all indicated that clinical laboratory indicators have guiding value for prognosis of cancer and also confirmed the reliability of the results derived from our analysis.

Although previous studies have attempted to systematically analyze the impact of laboratory data on the prognosis of SCLC, the main reference indicators were pathology information and treatment approaches, without incorporating complete laboratory data [43]. Another project has developed a nomogram model and included almost all laboratory data, but it was not a multicenter study [44]. Unlike other studies, we adopted the concept of omics to systematically evaluate the impact of almost all laboratory test data on prognosis, combined with clinical information, to perform a comprehensive analysis and employed a multicenter cohort to develop a prognostic model. Considering that the presence of censored data may have introduced bias into the model, and given that we only included 61 hematological laboratory indicators, which failed to capture some covariates that differ among patients with poor prognosis, these factors together led to the imperfect prediction of our model for patients with poor prognosis. Moreover, the small sample size is a limitation of this study. Future improvements could be made by increasing the number of patients in the cohort. Importantly, this model does indeed improve the accuracy of survival rate predictions compared to previous stage‐based models and is able to differentiate between different prognostic outcomes for patients in the same stage, which previous models did not achieve.

Considering that the laboratory indicators analyzed in this study have been used to construct a prognostic model and validated across multiple centers, which significantly improved the accuracy of prognosis predictions, it suggests to some extent that clinlabomics methods can provide valuable insights into clinical diseases and hold promise for future adoption.

## 4 Conclusions

The clinlabomics‐based nomogram model shows potential accuracy in predicting the prognosis of small cell lung cancer. By systematically evaluating blood laboratory data, it leverages existing resources to address clinical issues without increasing patient costs. This approach provides valuable prognostic information and may assist in risk assessment and stratification of newly admitted patients with small cell lung cancer, offering references for clinical decision‐making. In the next step, we plan to conduct a prospective study to evaluate the value of this model in the pre‐treatment prognostic risk assessment of patients with small cell lung cancer.

## 5 Methods

### 5.1 Patients Selection

Patient informed consent was waived and approved by the Institutional Ethics Committee of Sichuan Cancer Hospital (No. KY‐2021‐076) and The First Affiliated Hospital of Chengdu Medical College (2023CYFYIRB‐BA‐Oct06), respectively. All methods were carried out in accordance with relevant guidelines and regulations. The inclusion criteria were as follows: (1) confirmation of SCLC through histopathological methods; (2) completion of blood tests before initial treatment; and (3) availability of clinical follow‐up data. Exclusion criteria: (1) patients with concomitant severe diseases that could significantly impact prognosis; (2) patients lost to follow‐up or with incomplete or missing essential medical records. We retrieved all cases of small cell lung cancer from 2013 to 2020 through the Sichuan Cancer Hospital follow‐up system to avoid missing cases. A total of 196 cases were initially identified and finally included in this study. An external independent cohort also strictly followed the above process and enrolled 80 cases.

### 5.2 Patients Treatment

Both centers treat patients according to the National Comprehensive Cancer Network (NCCN) Clinical Practice Guidelines. For patients with T1‐2N0M0 stage limited‐stage small cell lung cancer, radical surgery and adjuvant platinum‐based chemotherapy are administered. For limited‐stage patients who are not candidates for surgery, concurrent radiation and platinum‐based chemotherapy are provided. Patients with extensive stage small cell lung cancer receive chemotherapy or combined immunotherapy based on chemotherapy. The chemotherapy regimens include carboplatin + etoposide, cisplatin + etoposide, irinotecan + carboplatin, irinotecan + cisplatin, paclitaxel + cisplatin, and paclitaxel + carboplatin.

### 5.3 Data Collection

The clinical data for the patients were obtained from the electronic medical record system of Sichuan Cancer Hospital and The First Affiliated Hospital of Chengdu Medical College. The primary information of patients in SCCH included demographic data (age, gender, smoking), pathological information (tumor location, AJCC staging), and laboratory test results (clinical biochemistry and basic clinical examinations conducted for all hospitalized patients). Another center only collected clinical pathological information and three types of test data from patients: TP, Lym ratio, AST. Abbreviations for all laboratory indicators referred to Table S1.

### 5.4 Statistical Analysis

Clinical laboratory data with missing values were imputed using the median. Since the laboratory blood indicators are part of the routine admission tests for small cell lung cancer patients, among the 61 items for 196 patients, HBsAg has the highest number of missing values, with 14 cases, accounting for 7.14%. The other items have very few missing values. Therefore, using median imputation has minimal impact on the overall data distribution of the entire project. The Kaplan–Meier method was used to calculate the relationship between test indicators and OS. The hazard ratio (HR) of the patient's survival curve was calculated using Cox regression. After correction with lasso regression, the Cox proportional hazards model was further used for screening. Variables with a *p* value of less than 0.01 in univariate analysis were included in multivariate regression. The training and validation groups in SCCH center were divided using time‐series grouping. The patients treated in SCCH from 2013.12 to 2017.04 were assigned to the training group. The rest were the validation group. After constructing the scoring model, the optimal cutoff value (with the smallest *p* value) was selected to differentiate between high‐risk and low‐risk groups. All statistical analyses were performed using R software (version 4.1.3), and a two‐sided *p* value of less than 0.05 was considered statistically significant.

## Author Contributions

**Qi Peng:** writing – original draft, investigation, methodology, software, visualization. **Fang Yang:** data curation, supervision, project administration, resources, visualization. **Ke Xu:** data curation. **Wei Guo:** data curation. **Dongsheng Wang:** conceptualization. **Mingfei Xiang:** data curation, supervision, project administration, resources. **Huaichao Luo:** conceptualization, project administration, supervision, resources, writing – review and editing, validation, formal analysis.

## Ethics Statement

The research related to human use has complied with all the relevant national regulations, institutional policies, and follows the tenets of the Helsinki declaration, and has been approved by the Institutional Ethics Committee of The Sichuan Cancer Hospital and The First Affiliated Hospital of Chengdu Medical College. The Institutional Review Board (IRB) of the Sichuan Cancer Hospital approved this retrospective study (IRB code: KY‐2021‐076) and the IRB of The First Affiliated Hospital of Chengdu Medical agreed to this study (Opinion letter Number: 2023CYFYIRB‐BA‐Oct06). This retrospective study has been approved to waive informed consent by the Institutional Review Board of the Sichuan Cancer Hospital and The First Affiliated Hospital of Chengdu Medical College.

## Consent

The authors have nothing to report.

## Conflicts of Interest

The authors declare no conflicts of interest.

## Supporting information


**Figure S1:** Validation of Novel program accuracy in multicenter cohort. (A) Receiver operating characteristic (ROC) curves for 2‐ and 3‐year survival in independent cohort. (B) Histogram of patient nomogram scores corresponding to clinical characteristics.

**Table S1:** Abbreviations for laboratory indicators. Table S2 Characteristics of 61 laboratory indicators of 196 patients in SCCH.

## Data Availability

The data that support the findings of this study are available on request from the corresponding author. The data are not publicly available due to privacy or ethical restrictions.
