# Supplementary material for: Development and Validation of a Clinlabomics‐Based Nomogram for Predicting the Prognosis of Small Cell Lung Cancer in China: A Multicenter, Retrospective Cohort Study
Source: Cancer Med. 2025 Aug 27;14(17):e71180. doi: 10.1002/cam4.71180 (PMC12381572; doi:10.1002/cam4.71180)
Supplement: Supplementary file 1 — Figure S1: Validation of Novel program accuracy in multicenter cohort. (A) Receiver operating characteristic (ROC) curves for 2‐ and 3‐year survival in independent cohort. (B) Histogram of patient nomogram scores corresponding to clinical characteristics. [file CAM4-14-e71180-s002.docx]

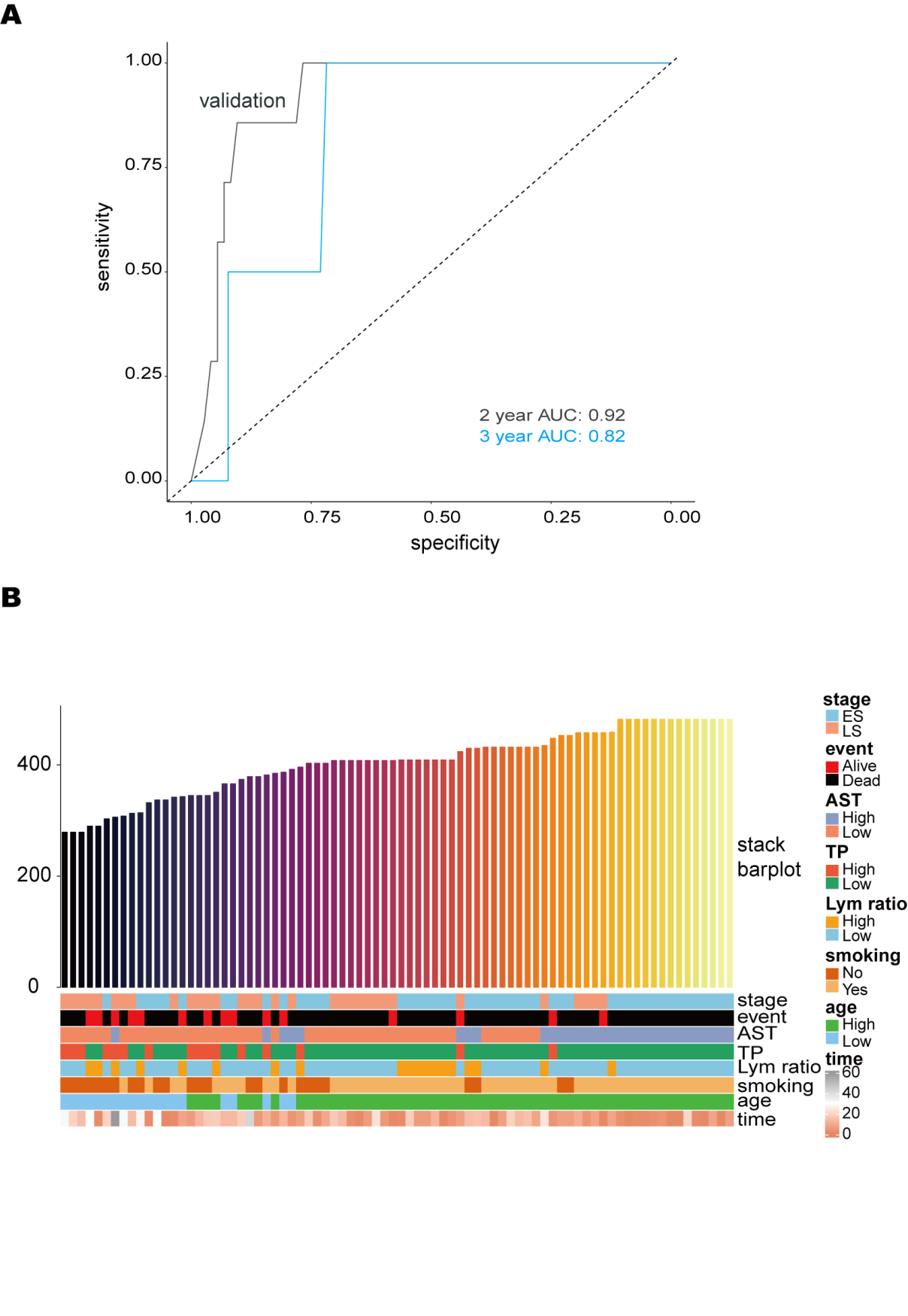


**Supplementary Fig.1 Validation of Novel program accuracy in multicenter cohort.**

**A** Receiver operating characteristic (ROC) curves for 2 and 3 years survival in independent cohort. **B** Histogram of patient nomogram scores corresponding to clinical characteristics.
