# Supplementary material for: Development and Validation of a Clinlabomics‐Based Nomogram for Predicting the Prognosis of Small Cell Lung Cancer in China: A Multicenter, Retrospective Cohort Study
Source: Cancer Med. 2025 Aug 27;14(17):e71180. doi: 10.1002/cam4.71180 (PMC12381572; doi:10.1002/cam4.71180)
Supplement: Supplementary file 2 — Table S1: Abbreviations for laboratory indicators. Table S2 Characteristics of 61 laboratory indicators of 196 patients in SCCH. [file CAM4-14-e71180-s001.docx]

**Supplementary Table 1 Abbreviations for laboratory indicators.**

| **Abbreviation** | **Full name** | **Abbreviation** | **Full name** |
| --- | --- | --- | --- |
| ALB | Albumin | N count | Neutrophil Count |
| Lym Ratio | Lymphocyte Ratio | TT | Thrombin Time |
| RBC | Red Blood Cell Count | HBsAb | Hepatitis B Surface Antibody |
| Ret high F intensity | Reticulocyte high Fluorescence intensity | Eos ratio | Eosinophil Ratio |
| Ret low F intensity | Reticulocyte low Fluorescence intensity | PLT | Platelet Count |
| Lym count | Lymphocyte Count | RDW-SD | Red Cell Distribution Width - Standard Deviation |
| TCH | Total Cholesterol | PT | Prothrombin Time |
| AST | Aspartate Aminotransferase | AFU | Alpha-L-Fucosidase |
| Hemoglobin | Hemoglobin | RDW-CV | Red Cell Distribution Width - Coefficient of Variation |
| MG | Magnesium ion | UA | Uric Acid |
| Pre-S1 antigen of HBV | Pre-S1 Antigen of Hepatitis B Virus | K | Potassium ion |
| Ret mid F intensity | Reticulocyte medium Fluorescence intensity | Bas count | Basophil Count |
| HCV Ag | Hepatitis C Virus Antigen | UREA | Blood Urea |
| D-D | D-Dimer | P | Phosphorus |
| Neu ratio | Neutrophil Ratio | ALT | Alanine Aminotransferase |
| HCV-Ab | Hepatitis C Virus Antibody | APTT | Activated Partial Thromboplastin Time |

**Table 1 (continued)**

| **Abbreviation** | **Full name** | **Abbreviation** | **Full name** |
| --- | --- | --- | --- |
| CA | Calcium ion | RET ratio | Reticulocyte Ratio |
| HBeAg | Hepatitis B e Antigen | Fibrinogen | Fibrinogen |
| MPV | Mean Platelet Volume | HBcAb | Hepatitis B Core Antibody |
| MCV | Mean Corpuscular Volume | CL | Chloride |
| TP-Ab | Treponema Pallidum Antibody | MPW | Mean Platelet Width |
| Bas ratio | Basophil Ratio | HBsAg | Hepatitis B Surface Antigen |
| TBIL | Total Bilirubin | CR | Creatinine |
| INR | International  Normalized Ratio | ALP | Alkaline Phosphatase |
| FDP | Fibrin Degradation Products | HBeAb | Hepatitis B e Antibody |
| CO2 | Carbon dioxide concentration | Mono count | Monocyte Count |
| Mono ratio | Monocyte Ratio | Ret count | Reticulocyte Count |
| DBIL | Direct Bilirubin | MCH | Mean Corpuscular Hemoglobin |
| TG | Triglycerides | Eos count | Eosinophil Count |
| GGT | Gamma-Glutamyl Transferase | PCT | Procalcitonin |

**Supplementary Table 2 Characteristics of 61 laboratory indicators of 196 patients in SCCH.**

| **Indicators** | **Median** | **Range** | **NA** |
| --- | --- | --- | --- |
| **HBcAb (PEIU/ml)** | 2.35 | (0-12.0) | 0 |
| **HCVAg (S/CO)** | 0.33 | (0.03-0.99) | 2 |
| **PreS1 antigen of HBV(mIU/ml)** | 0.99 | (0.02-31.17) | 0 |
| **TP-Ab(S/CO)** | 0.08 | (0.01-0.56) | 5 |
| **HBsAg(IU/ml)** | 10.66 | (0-231.87) | 14 |
| **HBsAb(mIU/ml)** | 96.47 | (0-960.0) | 0 |
| **HBeAg(IU/ml)** | 0.02 | (0-0.19) | 0 |
| **HBeAb(IU/ml)** | 1.11 | (0-6.30) | 0 |
| **PT(s)** | 10.90 | (9.30-16.60) | 12 |
| **INR** | 0.94 | (0.80-1.45) | 12 |
| **APTT(s)** | 29.70 | (17.40-55.10) | 12 |
| **TT(s)** | 3.53 | (13.0-21.0) | 12 |
| **FDP(ug/ml)** | 3.18 | (0-64.80) | 12 |
| **D-D(ug/ml)** | 0.33 | (0.09-13.39) | 12 |
| **MCV(fL)** | 94.05 | (60.80-105.90) | 0 |
| **MCH(pg)** | 30.65 | (18.50-35.20) | 0 |
| **RDW-CV(%)** | 13.25 | (11.50-16.80) | 0 |
| **RDW-SD(fL)** | 43.0 | (32.60-53.80) | 0 |
| **PLT(10^9/L)** | 197.5 | (73.0-472.0) | 0 |
| **PCT(%)** | 0.22 | (0.08-0.50) | 0 |
| **MPV(fL)** | 11.9 | (7.90-16.0) | 0 |
| **MPW** | 16.3 | (15.2-17.2) | 0 |
| **Ret count(10^12/L)** | 0.04 | (0.01-0.14) | 6 |
| **Neu ratio(%)** | 67.70 | (36.80-91.40) | 1 |
| **Lym ratio(%)** | 22.45 | (6.20-49.40) | 0 |
| **Mono ratio(%)** | 6.30 | (1.70-13.40) | 0 |
| **Eos ratio(%)** | 2.0 | (0-23.40) | 13 |
| **Bas ratio(%)** | 0.40 | (0-2.0) | 13 |
| **N count(10^9/L)** | 4.16 | (1.75-13.02) | 0 |
| **Lym count(10^9/L)** | 1.44 | (0.28-6.72) | 0 |
| **Mono count(10^9/L)** | 0.39 | (0.09-1.02) | 0 |
| **Eos count(10^9/L)** | 0.14 | (0-2.25) | 13 |
| **Ret ratio(%)** | 1.01 | (0.29-4.07) | 6 |
| **Bas count(10^9/L)** | 0.03 | (0-0.13) | 13 |
| **Ret Low F intensity(%)** | 95.65 | (71-100) | 6 |
| **Ret Mid F intensity(%)** | 4.35 | (0-19.20) | 6 |

**Table 2 (continued)**

| **Indicators** | **Median** | **Range** | **NA** |
| --- | --- | --- | --- |
| **Ret High F intensity(%)** | 0 | (0-9.80) | 6 |
| **RBC(10^12/L)** | 4.30 | (2.96-7.05) | 0 |
| **Hemoglobin(g/L)** | 131.0 | (81.0-177.0) | 0 |
| **CR(umol/L)** | 67.10 | (39.10-180.10) | 0 |
| **AST(U/L)** | 23.0 | (8.0-281.0) | 0 |
| **ALP(U/L)** | 85.0 | (38.0-394.0) | 0 |
| **GGT(U/L)** | 31.0 | (11.0-1393.0) | 0 |
| **TG(mmol/L)** | 1.22 | (0.40-4.63) | 0 |
| **UREA(mmol/L)** | 5.17 | (1.68-13.97) | 1 |
| **TCH(mmol/L)** | 4.69 | (2.42-7.21) | 0 |
| **CA(mmol/L)** | 2.31 | (1.12-3.20) | 0 |
| **MG(mmol/L)** | 0.97 | (0.57-1.25) | 0 |
| **TBIL(umol/L)** | 11.55 | (4.10-99.90) | 0 |
| **P(mmol/L)** | 1.11 | (0.51-1.72) | 0 |
| **K(mmol/L)** | 4.36 | (2.80-5.60) | 0 |
| **CL(mmol/L)** | 104.30 | (79.50-116.80) | 0 |
| **AFU(U/L)** | 22.80 | (8.0-69.20) | 0 |
| **CO2(mmol/L)** | 24.65 | (16.20-42.60) | 0 |
| **UA(umol/L)** | 301.50 | (72.0-642.0) | 0 |
| **DBIL(umol/L)** | 3.80 | (0.40-82.90) | 0 |
| **ALT(U/L)** | 21.50 | (6.0-276.0) | 0 |
| **TP(g/L)** | 67.25 | (52.0-84.30) | 0 |
| **ALB(g/L)** | 40.10 | (28.80-50.30) | 0 |
